# Quality of adverse event reporting in phase III randomized controlled trials of breast and colorectal cancer: A systematic review

**DOI:** 10.1002/cam4.3095

**Published:** 2020-05-26

**Authors:** Adam S. Komorowski, Helen J. MacKay, Rossanna C. Pezo

**Affiliations:** ^1^ Division of Medical Microbiology McMaster University Hamilton ON Canada; ^2^ Sunnybrook Research Institute Sunnybrook Health Sciences Centre Toronto ON Canada; ^3^ Division of Medical Oncology Sunnybrook Health Sciences Centre Toronto ON Canada; ^4^ Department of Medicine University of Toronto Toronto ON Canada

**Keywords:** adverse events, breast neoplasms, colorectal neoplasms, CONSORT, drug‐related side effects and adverse reactions, harms reporting, toxicities

## Abstract

**Background:**

Clinical trial reports often emphasize efficacy over harms, leading to misinterpretation of the risk‐to‐benefit ratio of new therapies. Clear and sufficiently detailed reporting of methods and results is especially important in the abstracts of trial reports, as readers often base their assessment of a trial on such information. In this study, we evaluated the quality of adverse event (AE) reporting and abstract quality in phase III randomized controlled trials (RCTs) of systemic therapies in breast and colorectal cancer.

**Methods:**

Medline, EMBASE, Cochrane Database of RCTs, and Cochrane Database of Systematic Reviews were searched from November 2005 to September 2018. Phase III RCTs evaluating systemic therapies in breast or colorectal cancer were included. Each article was independently reviewed by two investigators using a standardized data extraction form based on guidelines developed by the Consolidated Standards of Reporting Trials (CONSORT) group. Descriptive statistics, bivariate analysis, and multivariable linear regression were used to analyze data. All statistical tests were two‐sided.

**Results:**

Of 166 RCTs identified, 99.4% reported harms in the manuscript body, and 59.6% reported harms in the abstract. Reporting was restricted to severe harms in 15.6% of RCTs. Statistical comparison of AE rates went unreported in 59.0% of studies. Information regarding AEs leading to dose reductions, treatment discontinuations, or study withdrawals went unreported in 59.3%, 18.7%, and 86.8% of studies, respectively. Recently published RCTs (*P = *.009*)* and those sponsored at least partially by for‐profit companies (*P = *.003*)* had higher abstract quality scores.

**Conclusions:**

Breast and colorectal cancer phase III RCTs inadequately report CONSORT‐compliant AE data. Improved guideline adherence and abstract reporting is required to properly weigh benefits and harms of new oncologic therapies.

**Systematic Review Registration Number:**

CRD42019140673.

## Graphical abstract

Clinical trial reports often emphasize efficacy rather than toxicities of investigational agents, leading to misinterpretation of the risk‐to‐benefit ratio of new therapies. Here, we evaluated the quality of adverse event reporting in phase III randomized controlled trials of systemic therapies in breast and colorectal cancer. Our results show that most studies inadequately report adverse event data.

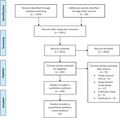

## 1 INTRODUCTION

Phase III randomized controlled trials (RCTs) assess the efficacy and harms of new treatment modalities in order to stringently determine their benefits and harms to patients. Accurate reporting is a necessary part of this determination: for example, inadequate reporting of adverse events (AEs) (harms) data can lead to misinterpretation of RCT results that may bias clinical decision‐making.^1^, ^2^, ^3^

The treatment landscape in oncology has shifted away from the cyclical use of cytotoxic chemotherapies—typically associated with episodic, severe toxicities of short duration—toward continuously administered targeted treatments that may produce chronic, lower grade, and multi‐organ system toxicities. As a result of increasingly long‐term use of therapies, stringent AEs reporting has become more necessary. In addition, increasing sample sizes in oncologic RCTs of novel therapies has allowed the detection of smaller treatment effects^4^, ^5^

The Consolidated Standards of Reporting Trials (CONSORT) statement was introduced in 2001 as a way to standardize reporting in RCTs.^6^ The CONSORT Harms Checklist was published in November 2004 to improve the reporting of AEs in order to foster both increased transparency and consistency of harms reporting in RCTs.^7^

In 2008, Hopewell *et al* published a CONSORT reporting checklist for RCT abstracts in the hopes of encouraging more stringent reporting of harms.^8^ This checklist takes into account the following: trial title, design description, eligibility criteria, interventions, specific hypothesis, primary outcome definition, description of randomization and blinding, number of patients randomized, trial status, number of patients analyzed, primary outcome effect size and precision, harms, general interpretation of the results, trial registration number, and source of funding. Seeing as many researchers glean trial information from an abstract, a clear set of harms reporting items specifically for abstracts is a vital extension of the CONSORT statement.^8^

Unfortunately, adherence to the CONSORT reporting items remains suboptimal to the present day. Pitrou *et al* examined the reporting of safety results from the general medical literature in 2009, finding that 27.1% of studies analyzed did not provide information on severe AEs, and 47.4% did not provide information on withdrawal of patients due to an AE.^9^ In the general medical literature, multiple studies have demonstrated that only half of phase III RCT abstracts report harms in an appropriate manner.^10^, ^11^, ^12^ This trend has been echoed in other specialties, including critical care.^13^ Examining metastatic solid tumor phase III RCT abstracts, Sivendran *et al* showed 74% reported serious or unexpected AEs,^14^ while Ghimire *et al* demonstrated a 77% adherence to harms reporting in oncology phase III RCT abstracts.^15^

The primary aim of this study was to systematically review and evaluate the quality of AE reporting in phase III breast and colorectal cancer RCTs. We also examined whether specific trial characteristics were associated with the quality of AE reporting, and could be predicted by an abstract quality score.

## 2 MATERIALS AND METHODS

### 2.1 Data sources

Medline, EMBASE, the Cochrane Database of Randomized Controlled Trials (CCRCT), and the Cochrane Database of Systematic Reviews (CDSR) were systematically searched in duplicate from November 2005 to 14 September 2018 using subject headings and keywords to capture breast cancer, colorectal cancer, AEs, and RCT terms (Breast Neoplasms; Colorectal Neoplasms; clinical trial, phase III; adverse drug reaction). The initial time point of November 2005 was chosen to capture studies published one calendar year after the introduction of the CONSORT AE reporting guidelines in November 2004. Subject headings and keywords were modified for each database according to its unique indexing terms. The search (Data S1) was conducted by a medical information specialist and limited to humans, with English language restrictions. Grey literature and reference lists of retrieved articles were also screened for additional relevant studies.

### 2.2 Study selection

This review was conducted and reported according to PRISMA guidelines,^16^ and the study protocol was registered with the PROSPERO International Prospective Register of Systematic Reviews (CRD42019140673). The aim of this review was to examine the quality of AEs reporting in phase III RCTs of systemic therapies in breast and colorectal cancer. Thus, all phase III RCTs evaluating drug regimens (chemotherapy, endocrine therapy, immunotherapy, and targeted agents) in breast cancer or colorectal cancer patients were included. Studies were excluded if the patient population did not include breast cancer or colorectal cancer patients; if trials were evaluating surgical or radiotherapeutic treatment modalities; or if duplicate data were reported. Phase I, II, and IV RCTs, editorials, commentaries, reviews, cohort studies, and case‐control studies were excluded. Reference lists of excluded studies were screened to identify any potentially relevant studies. One reviewer (ASK) selected potentially eligible studies by independently screening titles and abstracts of identified studies. Full texts of the studies identified were subsequently retrieved and independently assessed for eligibility by one reviewer (ASK).

### 2.3 Data extraction

A data extraction form (Data S2) was developed based on the CONSORT Recommendations, as well as the CONSORT Harms Checklist.^6^, ^7^ The form was modified from a previously published checklist used to evaluate the quality of harms reporting in the general medical literature.^9^ The data extraction form was designed to capture information from the both the abstract and the entire clinical trial report, and was divided into the following sections: methodology, sponsorship, results, reporting of AEs, reporting of severity, reporting of need for treatment discontinuations (TDs) and dose reductions (DRs), and reporting of statistical tests for AEs. AE data described in appendices or supplementary files were considered to be part of the “full text” of the included studies for the purposes of this review. Data were extracted independently by two reviewers (ASK and RCP).

During data extraction, an abstract quality score was assigned to each publication that took into account the following reporting items: explanation of study rationale; brief description of participants; description of intervention; explicit statement of primary endpoint; duration of follow‐up; reporting of planned sample size; *p*‐value or confidence interval reporting; description of AEs or toxicities; and specification of funding source.

### 2.4 Risk of bias assessment

The Cochrane Risk of Bias Tool for Randomized Controlled Trials 2.0 (RoB 2.0) was deemed appropriate for use in assessing the included studies, given that the studies under systematic review were RCTs. The RoB 2.0 rates studies as “low risk”, “unclear risk”, or “high risk” of bias using preestablished criteria to evaluate both study design and applicability.^17^ Risk of bias was determined by a single reviewer (ASK).

### 2.5 Primary and secondary outcomes

The primary outcome was the evaluation of the quality of AE reporting according to the percentage of trials including detailed information on AEs (reporting of AEs in figure/table vs only text, per treatment arm, separation of expected/unexpected AEs, and scale used for AE severity) of dose reduction and treatment discontinuations in phase III RCTs of breast and colorectal cancer treatment regimens. The secondary outcome was based on an exploratory analysis of trial characteristics, with the aim of determining whether specific trial characteristics were associated with the quality of the abstract, as represented by a numerical score assigned during data extraction (see “Materials and Methods—Data Extraction”).

### 2.6 Statistical analysis

Statistical analysis was performed by a biostatistician using SAS 9.4 (Cary, SAS Institute). Descriptive statistics, including mean and standard deviation, were used for continuous variables, whereas categorical variables were described with frequencies and percentages and compared using the chi‐squared test. All statistical tests were two‐sided, and statistical significance was defined as *P < *.05. Bivariate linear analysis was used to examine the association between the abstract quality score assigned during data extraction and selected trial characteristics. Significant covariates found during bivariate analysis were used to run the multivariable linear regression model. Multivariable linear regression analysis was thus used to identify trial characteristics associated with inadequate safety reporting. Given the lack of sufficient homogeneity between included studies with regards to participants, interventions, and outcomes, a meaningful summary statistic could not be calculated; the clinical and methodological heterogeneity obviated the use of meta‐analysis on these studies.

## 3 RESULTS

### 3.1 Study characteristics

**FIGURE 1:**
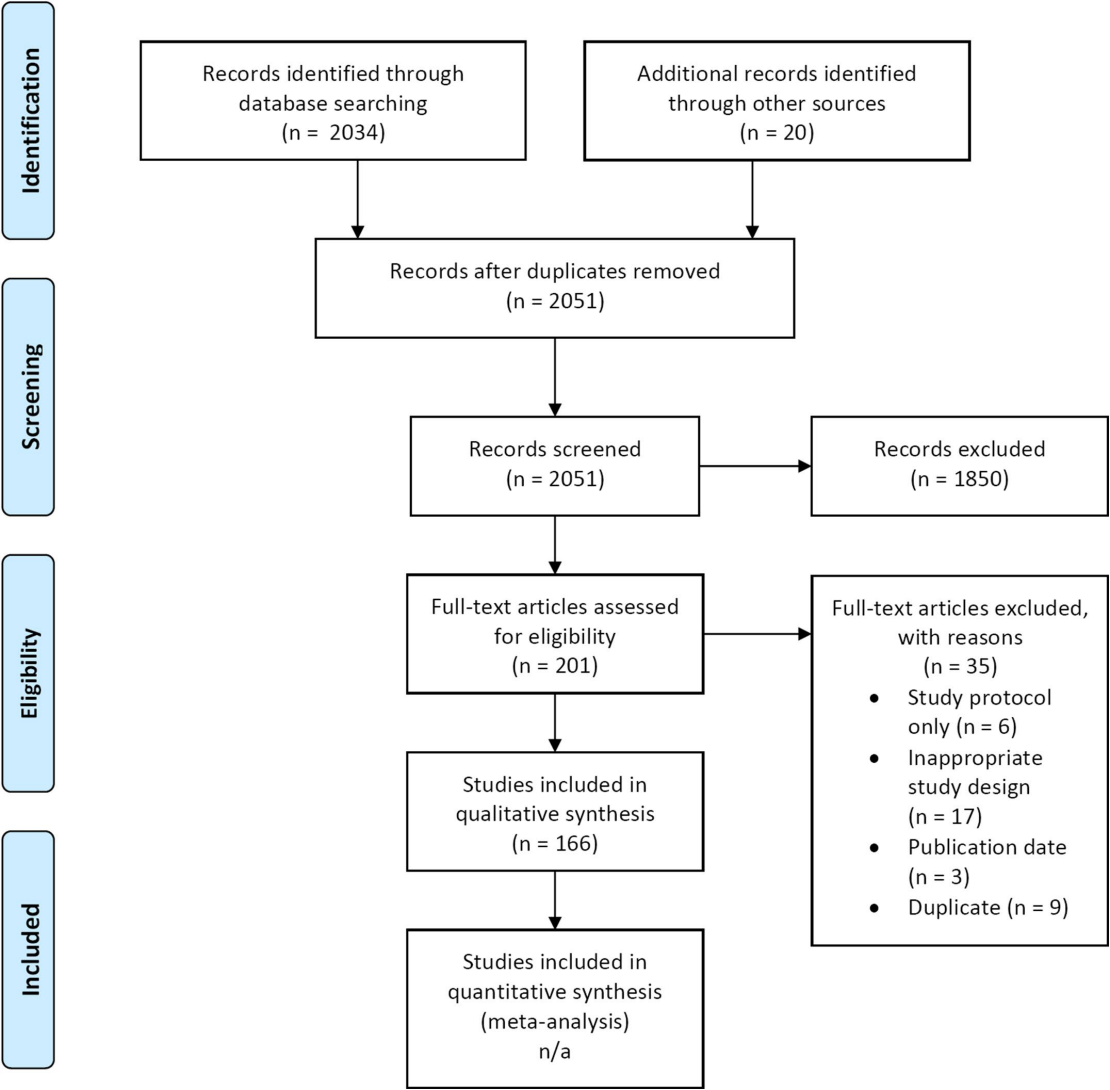
PRISMA flow diagram

The literature search yielded 2034 abstracts; an additional 20 studies were identified through grey literature searches. After removal of duplicates and assessment by reviewers, 201 full texts were assessed for inclusion. 35 full texts were excluded from analysis, with reasoning provided in Data S3. Ultimately, 166 studies were included in this systematic review (Figure 1).^18^, ^19^, ^20^, ^21^, ^22^, ^23^, ^24^, ^25^, ^26^, ^27^, ^28^, ^29^, ^30^, ^31^, ^32^, ^33^, ^34^, ^35^, ^36^, ^37^, ^38^, ^39^, ^40^, ^41^, ^42^, ^43^, ^44^, ^45^, ^46^, ^47^, ^48^, ^49^, ^50^, ^51^, ^52^, ^53^, ^54^, ^55^, ^56^, ^57^, ^58^, ^59^, ^60^, ^61^, ^62^, ^63^, ^64^, ^65^, ^66^, ^67^, ^68^, ^69^, ^70^, ^71^, ^72^, ^73^, ^74^, ^75^, ^76^, ^77^, ^78^, ^79^, ^80^, ^81^, ^82^, ^83^, ^84^, ^85^, ^86^, ^87^, ^88^, ^89^, ^90^, ^91^, ^92^, ^93^, ^94^, ^95^, ^96^, ^97^, ^98^, ^99^, ^100^, ^101^, ^102^, ^103^, ^104^, ^105^, ^106^, ^107^, ^108^, ^109^, ^110^, ^111^, ^112^, ^113^, ^114^, ^115^, ^116^, ^117^, ^118^, ^119^, ^120^, ^121^, ^122^, ^123^, ^124^, ^125^, ^126^, ^127^, ^128^, ^129^, ^130^, ^131^, ^132^, ^133^, ^134^, ^135^, ^136^, ^137^, ^138^, ^139^, ^140^, ^141^, ^142^, ^143^, ^144^, ^145^, ^146^, ^147^, ^148^, ^149^, ^150^, ^151^, ^152^, ^153^, ^154^, ^155^, ^156^, ^157^, ^158^, ^159^, ^160^, ^161^, ^162^, ^163^, ^164^, ^165^, ^166^, ^167^, ^168^, ^169^, ^170^, ^171^, ^172^, ^173^, ^174^, ^175^, ^176^, ^177^, ^178^, ^179^, ^180^, ^181^, ^182^, ^183^ Characteristics of included studies are summarized in Table 1, whereas the complete set of consensus extracted data may be found in Data S4A (the data extraction set of Reviewer 1 is found in Data S4B*,* and the data extraction set of Reviewer 2 is found in Data S4C). All studies were published on or after November 2005, inclusive. The included studies cover each year of the date range captured by the search strategy, and represent diverse patient populations from North America, Europe, Asia, and Australia. Of the included studies, 121 (72.9%) evaluated any‐stage breast cancer, whereas 45 (27.1%) evaluated any‐stage colorectal cancer. Eighteen studies (10.8%) investigated neoadjuvant treatment regimens, whereas 51 (30.7%), 54 (32.5%), and 43 (25.9%) studies evaluated adjuvant, first‐line metastatic, and ≥second‐line metastatic treatment regimens, respectively. The median sample size of included studies was 627 patients (range: 51‐9779). A total of 138 included studies (83.1%) were at least partially funded by industry; however, only 38 studies (22.9%) explicitly stated provision of study drug by a for‐profit sponsor.

**TABLE 1:** Characteristics of included studies (n = 166)

	Number of trials (n)	Percentage (%)
Year of publication		
2005	3	1.81
2006	4	2.41
2007	9	5.42
2008	1	0.60
2009	9	5.42
2010	6	3.61
2011	8	4.82
2012	11	6.63
2013	12	7.23
2014	15	9.04
2015	10	6.02
2016	35	21.08
2017	25	15.06
2018	18	10.84
Results of primary outcome		
Positive	100	60.2
Negative	65	39.2
Lead trial region		
North America	40	24.1
Europe	86	51.8
Asia	33	19.9
Other	7	4.2
Sponsorship		
For profit	100	60.2
Non‐profit	21	12.7
Mixed	38	22.9
Not stated	7	4.2
Study drug provided by for profit company		
Yes	38	22.9
No	12	7.2
Not reported	116	69.9
Tumor site		
Breast	121	72.9
Colorectal	45	27.1
Line of therapy		
Neoadjuvant	18	10.8
Adjuvant	51	30.7
Metastatic, first‐line	54	32.5
Metastatic, ≥second‐line	43	25.9
Primary outcome		
Disease‐free survival (DFS)	20	12.0
Progression‐free survival (PFS)	61	36.8
Overall survival (OS)	29	17.5
Time to progression (TTP)	8	4.8
Other	54	32.5
Type of investigational therapy		
Cytotoxic chemotherapy	56	33.7
Endocrine therapy	19	11.4
Targeted therapy	19	11.4
Combination of cytotoxic chemotherapy and endocrine therapy	3	1.8
Combination cytotoxic chemotherapy and targeted therapy	49	29.5
Other	20	12.0
Sample size (number of patients)		
Median	627	
Mean	1089.95	
Range	51‐9779	

### 3.2 Safety reporting

The main results of the harms reporting analysis are described in Table 2. Of the studies examined, 165 (99.4%) reported AEs in the body of the manuscript, and the majority of reports had at least one table/figure to report AEs (n* = *159*,* 95.8%). While most (n* = *162, 97.6%) manuscripts described AEs per arm, four included studies described AEs in only one arm. Severe AEs (typically greater than grade 3 on the Common Terminology Criteria for Adverse Events scale) were the sole reported type of AE in 26 studies (15.6%). Notably, a majority of studies (n* = *98*,* 59.0%) did not perform a statistical comparison of AE rates between study arms.

**TABLE 2:** Presentation of adverse events, dose reductions, and treatment discontinuation in the results section of trial publications (n = 166)

	No. of trials (n)	Percentage (%)
Mode of presentation of adverse events (AEs)		
Figure/Table	159	95.8
Text	165	99.4
Reported AEs per arm	162	97.6
Reported AEs in only one arm	4	2.4
Only severe AEs reported	26	15.6
Separation of expected/unexpected AEs		
Yes	4	2.4
No	162	97.6
Statistical comparison of AE rates between study arms		
Yes	68	41.0
No	98	59.0
Scale used to report AE severity		
NCI CTCAE (all versions)	143	86.1
WHO	6	3.6
Other	3	1.8
No scale or unknown	14	8.4
Reporting of AEs leading to dose reductions	67	40.7
Reporting of AEs leading to treatment discontinuation	135	81.3
Reporting of AEs leading to withdrawal from study	22	13.2
Reporting of deaths due to AEs	108	65.4

A scale for severity grading of AEs was identified in 91.5% (n* = *152) of trials. The most commonly used scale—in 143 trials (86.1%)—was the Common Terminology Criteria for Adverse Events (CTCAE). A small number of trials (n* = *14*,* 8.4%) omitted a severity scale or failed to report which scale was used. Notably absent from many of the included studies was information on AEs leading to dose reductions, treatment discontinuations, study withdrawal, or death. AEs leading to dose reduction or treatment discontinuation went unreported in 59.3% and 18.7% of studies, respectively. AEs leading to study withdrawal were not reported in 86.8% of studies, and no information on deaths due to AEs was reported in 34.6% of trials.

### 3.3 Abstract quality scoring

Linear regression of exploratory variables using abstract quality score as the dependent variable identified several significant trends outlined in Tables 3 and 4.

**TABLE 3:** Results of regression analyses of trial characteristics predictive of abstract quality score

			Regression analysis					
Trial characteristic		Abstract quality score (0‐10)	Bivariate analysis			Multivariable analysis		
		Mean	Regression coefficient estimate	*t*	*P*	Regression coefficient estimate	*t*	*P*
Year of publication, continuous		6.8	.09604	3.61	.0004	.08837	3.39	.0013
Results of primary outcome								
Negative		7.1	−.115	−0.58	.5645	Not investigated in model		
Positive		7.2	n/a	n/a	n/a	Not investigated in model		
Sponsorship								
Non‐industry		6.9	n/a	n/a	n/a	n/a	n/a	n/a
Industry		7.3	.78786	3.03	.0028	.82252	3.17	.0019
Mixed		7.5	.97838	3.23	.0015	.88939	3.01	.0031
Intent of study therapy								
Curative	7.2		n/a	n/a	n/a	.31387	1.63	.1050
Palliative	7.1		n/a	n/a	n/a	n/a	n/a	n/a
Tumor site								
Breast		7.2	n/a	n/a	n/a	Not investigated in model		
Colorectal		7.1	−.1107	−0.51	.6139	Not investigated in model		
Line of therapy								
Neoadjuvant		6.9	7.33721	−1.12	.2627	Not investigated in model		
Adjuvant		7.4	.05495	0.21	.8314	Not investigated in model		
Metastatic, first line		6.9	−.402	−1.58	.116	Not investigated in model		
Metastatic, ≥ second line		7.3	n/a	n/a	n/a	Not investigated in model		
Type of investigational therapy								
Cytotoxic chemotherapy		7.0	−.0214	−0.07	.9475	Not investigated in model		
Endocrine therapy		7.0	−.0487	−0.12	.9031	Not investigated in model		
Targeted therapy		7.8	.76711	1.92	.056	Not investigated in model		
Combination cytotoxic chemotherapy and endocrine therapy		7.0	−.075	−0.1	.9227	Not investigated in model		
Combination cytotoxic chemotherapy and targeted therapy		7.2	.10867	0.33	.7429	Not investigated in model		
Other		7.1	n/a	n/a	n/a	Not investigated in model		

**TABLE 4:** Overall *P*‐values for regression analyses of trial characteristics predictive of abstract quality score

Trial characteristic	Overall *P*‐values	
	Bivariate analysis	Multivariable analysis
Intent of study therapy	.0034	0.0040
Line of therapy	.1386	Not investigated in model
Type of investigational therapy	.2724	Not investigated in model

The results of bivariate analysis demonstrated that a recent year of publication was significantly associated with a high‐abstract quality score (*β* = .096, *t* = 3.61, *P = *.0004). In addition, the provision of the study drug by a for‐profit sponsor was significantly associated with a high‐abstract quality score (*β* = .56, *t* = 2.48, *P = *.014*)*.

The overall model fit for the final multivariable regression equation was *R* ^2^ = .14. Papers published in recent years had significantly higher abstract quality scores (*P = *.009). Compared with papers that received nonprofit sponsorship, those with either for‐profit (*P = *.002*)* or mixed sponsorship (*P = *.003) had significantly higher abstract quality scores. There was no statistically significant difference between the abstract quality scores of breast and colorectal cancer RCTs that were investigating curative vs palliative treatment regimens (*P = *.10*)*.

### 3.4 Risk of bias

**FIGURE 2:**
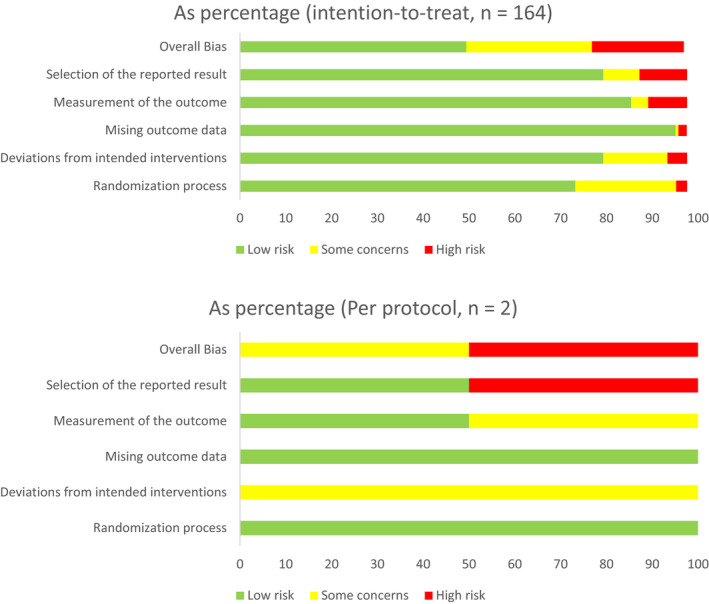
Risk of bias summary of included studies

Of the included studies (n = 166), 164 were analyzed for risk of bias using the RoB 2.0 intention‐to‐treat checklist, while two papers were analyzed using the per‐protocol checklist. Of the 164 intention‐to‐treat studies, 49.4% were deemed overall to have a low risk of bias, 27.4% were deemed to have an unclear risk of bias, and 20.1% were deemed to have a high risk of bias. (Figure 2)(For the complete risk of bias data, see Data S5).

Of the two per‐protocol studies, one had an unclear risk of bias, and one had a high risk of bias. Both studies had a low risk of bias in the “randomization process” and “missing outcome data” domains; both studies had an unclear risk of bias in the “deviation from intended interventions” domain; 50% had a low, and 50% had an unclear, risk of bias in the “outcome measurement” domain; finally, 50% had a low, and 50% had a high risk of bias in the “selection of reported result” domain.

## 4 DISCUSSION

In this systematic review, we evaluated the quality of AE reporting according to the CONSORT guidelines in phase III RCTs of drug regimens in breast and colorectal cancer patients between November 2005 and September 2018. Most studies do not adequately report harms as per the CONSORT guidelines. Although virtually all studies analyzed reported AEs in the main manuscript text (n* = *165*,* 99.4%), 40.4% of reports either inadequately reported or did not include harms‐related results in the study abstract. A 2009 analysis of trial abstracts by Berwanger *et al* in four major medical journals found that only half reported AE data in their abstracts.^10^ Another study in 2013 reiterated the finding of substandard AE reporting, with only 32% of breast cancer RCT abstracts adequately reporting harms.^184^ The CONSORT authors and other groups have acknowledged the utility of stating AEs in phase III RCT abstracts: this data is important for not only establishing databases, but also retrieving appropriate information used in clinical decision‐making.^7^, ^185^

Severe AEs (typically greater than grade 3 on the Common Terminology Criteria for Adverse Events scale) were the sole reported type of AE in 26 studies (15.6%). Lower grade toxicities that are persistent over a prolonged period may be intolerable for patients if they have a negative impact on quality of life. For example, small molecule inhibitors—which are often offered in daily dosage regimens for some cancers—and the presence of chronic, low‐grade toxicities, may limit adherence to these treatments in routine practice. A lack of adherence to therapy would minimize the benefits observed in RCTs. Indeed, studies themselves may select for patient populations that underestimate the impact of low‐grade toxicities: eligible patients for RCTs typically have fewer comorbidities and higher performance statuses. Furthermore, the supportive care available to study patients may help mitigate the impact of low‐grade AEs on quality of life in a way that is not reproducible in routine practice. For example, clinical trial nurses, dosing diaries, structured dose modification criteria, and trial educational materials/programs all offer support outside typical practice norms. Thus, the sole reporting of severe AEs may severely limit the ability of oncologists to provide appropriate counsel to patients, mitigating their ability to provide supportive care when needed.

Among the studies we systematically reviewed, reporting of dose modification due to AEs was poor: dose reductions went unreported in 59.3%. These high figures represent a key flaw in the way most studies report their findings. Accurate reporting of dose reductions is important; it reflects whether the starting dose that is chosen for a RCT is appropriate and tolerable. An investigational agent that may be associated with few grade 3 or higher toxicities—but frequent low‐grade toxicities—may not be tolerable with prolonged administration. The recommended phase II dose (RP2D) and schedule of administration for an investigational agent is established during dose escalation phase I trials. There are a limited number of patients treated at the RP2D during phase I/II trials: these patients may not reflect the patient population enrolled in an RCT, or those who are treated outside of clinical trials in routine practice once an investigational agent has been approved by a regulatory body. Patients enrolled in phase I/II studies usually have advanced disease previously treated with multiple lines of prior therapy; they may only be treated an investigational agent for a short period of time, usually less than 6 months, and may be more willing to accept low‐grade toxicities that patients exposed to few lines of therapy, or with early stage disease treated in the adjuvant setting. In addition, phase I/II trials are usually performed by a small number of investigators who may be more experienced with toxicity management than phase III RCTs. In the adjuvant setting, patients may be less tolerable of low‐grade toxicities. Furthermore, for the palliative management of metastatic disease, the burden of harm and its often profound impact on quality of life must be balanced against improvements in disease‐related symptoms and survival.

Less than half (41.0%) of the RCTs examined included statistical comparisons of AEs between treatment arms. In addition, many studies did not identify the population used for safety analysis. Identifying the population used for safety analysis is likewise necessary, as the exclusion of any treated patients could bias the interpretation of harms‐related reporting.

Similar findings to those observed in our study of breast and colorectal cancer RCTs have been reported in other medical disciplines. In the general medical literature, Pitrou *et al* found that 18% of reports did not describe AEs with numerical data, and that information relating to the withdrawal of patients due to AEs was missing in 47% of papers.^9^ In our study, we found that 18.7% of papers did not give information on the need to discontinue treatment due to AEs, whereas data on patient withdrawal due to AEs were missing in 86.8% of papers. Our study was also concordant with analyses in the oncology literature: for example, a 2016 paper by Maillet *et al* that reviewed oncology RCTs between 2007 and 2011 indicated that frequency and nature of grade 5 AEs were adequately reported in 50%, AEs leading to study withdrawal in 19%, and AEs leading to dose reduction in 13% of manuscripts.^186^

Using bivariate and multivariable linear regression in an exploratory analysis, we examined whether any study characteristics were associated with the abstract quality scores we assigned during data extraction. Both bivariate and multivariable analyses showed that a recent year of publication was associated with a higher abstract quality score (*P = *.009*,* multivariable model).

We also examined whether industry‐sponsored studies had better reporting of AEs. An earlier study from the neurology literature of antiepileptic RCTs described a poor quality of AE reporting,^187^ with improved safety reporting in studies sponsored by for‐profit companies, compared with studies having an academic hospital or cooperative group sponsor. Our study similarly found that industry‐sponsored studies tended to have improved AE reporting than those sponsored by nonprofit groups: those with either for‐profit (*P = *.002*)* or mixed sponsorship (*P = *.003) had significantly higher abstract quality scores. This may be due to the added costs of collecting detailed data on AEs, or possibly as a result of guidelines on data collection and reporting in pharmaceutical‐sponsored studies.

### 4.1 Limitations

A limitation of this study was that the search strategy was limited to English‐language publications; however, as there were no other restrictions and a large number of studies were included, this is unlikely to compromise this review's integrity. Overall, selection and performance biases were moderate across studies, while detection and attrition biases were generally low. The impact of selection and performance biases on the conclusions of this review is minimized by restricting the multivariable analysis to determining how well study abstracts report AEs data. A further limitation of this study is the lack of analysis of quality of life data which would allow situating our study more deeply in the patient‐centric experience.

## 5 CONCLUSION

Our systematic review highlights the incomplete reporting of harms in breast and colorectal cancer RCTs. A more complete description of harms is needed in order to better understand the therapeutic index of new treatments. We propose that adherence to the CONSORT AE statement should be a mandatory requirement of phase III RCT publication in medical journals, in order to ensure consistent reporting of harms data across trials. With the increasing use of immunotherapies and targeted therapies, oncologic RCTs in general may also require additional standards for the reporting of low‐grade toxicities that lead to dose interruptions, dose reductions, and treatment discontinuations. Such reporting standards may help indicate the tolerability of investigational agents administered over a long period of time and would move the RCT investigational paradigm closer to a more holistic, patient‐centered view of clinical outcomes.

## CONFLICT OF INTEREST

The authors report no relevant conflicts of interest.

## AUTHOR CONTRIBUTIONS

Conception, design, collection of data, analysis, interpretation: ASK and RCP; writing, review and revision of manuscript: ASK and RCP. Funding acquisition and oncology expertise: HJM and RCP.

## Supporting information

Data S1

Data S2

Data S3

Data S4A

Data S4B

Data S4C

Data S5

## Data Availability

Source data are available from the corresponding author on reasonable request.
