# Supplementary material for: Quality of adverse event reporting in phase III randomized controlled trials of breast and colorectal cancer: A systematic review
Source: Cancer Med. 2020 May 26;9(14):5035–50. doi: 10.1002/cam4.3095 (PMC7367648; doi:10.1002/cam4.3095)
Supplement: Supplementary file 1 — Data S1 [file CAM4-9-5035-s001.docx]

**Supplementary Data S.1**

**Search Strategy and Summary of Results**

| **Database** | **Time Period** | **Results (*n*)** |
| --- | --- | --- |
| Medline | 16 Nov 2005 - 14 Sept 2018 | 1479 |
| EmBASE | 16 Nov 2005 - 14 Sept 2018 | 219 |
| CCRCT | 16 Nov 2005 - 14 Sept 2018 | 322 |
| CDSR | 16 Nov 2005 - 14 Sept 2018 | 12 |
| **Total** | | **2029** |

**Medline Search Strategy**

1. exp Breast Neoplasms/
2. cecal neoplasms/ or colorectal neoplasms/ or exp colonic neoplasms/ or colorectal neoplasms, hereditary nonpolyposis/ or rectal neoplasms/
3. 1 or 2
4. clinical trial, phase iii/ or randomized controlled trial.mp.
5. clinical trials, phase III as topic/ or randomized controlled trials as topic/
6. phase iii.mp,kw.
7. phase 3.mp,kw.
8. or/4-7
9. 3 and 8
10. exp animals/ not (exp humans/ and exp animals/)
11. 9 not 10
12. limit 11 to (consensus development conference or consensus development conference, nih or guideline or meta analysis or news or newspaper article or "review" or "scientific integrity review" or systematic reviews)
13. 11 not 12
14. limit 13 to (english language and yr="2005 - Current")

**EmBASE Search Strategy**

1. exp breast cancer/
2. exp colon cancer/
3. 1 or 2
4. exp chemotherapy/
5. adverse drug reaction/
6. (adverse event* or harms reporting).mp
7. 5 or 6
8. randomized controlled trial/
9. phase iii.mp,kw.
10. phase 3.mp,kw.
11. 9 or 10
12. 8 and 11
13. 3 and 4 and 7 and 12
14. limit 13 to (Human and English language and yr="2005 - Current")

**Cochrane Database of Systematic Reviews (CDSR) Search Strategy**

1. (breast cancer or colon cancer or breast neoplasm* or colon neoplasm*).mp
2. chemotherapy.mp
3. (adverse event* or adverse reaction* or harms reporting).mp
4. randomized controlled trial.mp.
5. (phase iii or phase 3).mp.
6. 4 and 5
7. 1 and 2 and 3 and 6
8. limit 7 to last 17 years

**Cochrane Database of Randomized Controlled Trials (CCRCT) Search Strategy**

1. exp Breast Neoplasms/
2. colon cancer.mp.
3. exp Colonic Neoplasms/
4. or/1-3
5. chemotherapy.mp. or exp Drug Therapy/
6. exp "Drug-Related Side Effects and Adverse Reactions"/
7. (adverse event* or harms reporting).mp.
8. 6 or 7
9. clinical trial, phase iii/ or randomized controlled trial.mp
10. clinical trials, phase III as topic/ or randomized controlled trials as topic/
11. phase iii.mp,kw.
12. phase 3.mp,kw.
13. or/9-12
14. 4 and 5 and 8 and 13
15. exp animals/ not (exp humans/ and exp animals/)
16. 14 not 15
17. limit 16 to (yr="2005 - Current" and English language)
