# Supplementary material for: Quality of adverse event reporting in phase III randomized controlled trials of breast and colorectal cancer: A systematic review
Source: Cancer Med. 2020 May 26;9(14):5035–50. doi: 10.1002/cam4.3095 (PMC7367648; doi:10.1002/cam4.3095)
Supplement: Supplementary file 2 — Data S2 [file CAM4-9-5035-s002.docx]

**Data Extraction Form**

Study Origin ____________

## Study Methodology

1. Abstract: ⁪ structured^1^ ⁪ not structured^2^

| **Item** | **Yes** | **Partial** | **No** |
| --- | --- | --- | --- |
| INTRODUCTION |  |  |  |
| 1. rationale for study explained ………………………….. |  |  |  |
| METHODS |  |  |  |
| 2. intervention described in sufficient detail …………….. |  |  |  |
| 3. primary endpoint explicitly stated …………………….. |  |  |  |
| 4. duration of follow-up stated ……………………………. |  |  |  |
| 5. planned sample size reported ………………………… |  |  |  |
| 6. p-values or confidence interval reported …………….. |  |  |  |
| RESULTS |  |  |  |
| 7. brief description of participants ……………………….. |  |  |  |
| 8. size of treatment effect reported ……………………… |  |  |  |
| 9. adverse events/ toxicity described …………………… |  |  |  |
| 10. source of funding specified |  |  |  |

Abstract Quality Score: /10

Study outcome: ⁪positive^1^ ⁪ negative^2^

1. Disease site: ⁪ breast^1^ ⁪ colorectal^2^ ⁪colon^3^ ⁪rectal^4^ ⁪ prostate^5^
2. Setting: ⁪ adjuvant^1^ ⁪ neoadjuvant^2^ ⁪ metastatic^3^ ⁪ surgical^4^

Line of treatment (if metastatic): ⁪ first^1^ ⁪ second or more^2^ ⁪ N/A^3^

1. Primary end-point:

⁪ abstract

⁪ explicit^1^ ⁪ implied^2^ ⁪ not stated^3^

⁪ OS^4^ ⁪ DFS^5^ ⁪ PFS^6^ ⁪ TTP^7^ ⁪ other^8^ _______

⁪ paper

⁪ explicit^1^ ⁪ implied^2^ ⁪ not stated^3^

⁪ OS^4^ ⁪ DFS^5^ ⁪ PFS^6^ ⁪ TTP^7^ ⁪ other^8^ _______

1. Secondary end-point:

⁪ abstract

⁪ explicit^1^ ⁪ implied^2^ ⁪ not stated^3^

⁪ OS^4^ ⁪ DFS^5^ ⁪ PFS^6^ ⁪ TTP^7^ ⁪ other^8^ _______

⁪ paper

⁪ explicit^1^ ⁪ implied^2^ ⁪ not stated^3^

⁪ OS^4^ ⁪ DFS^5^ ⁪ PFS^6^ ⁪ TTP^7^ ⁪ other^8^ _______

1. Control group: placebo^1^ BSC^2^ ⁪ active agent/other treatment^3^ observation^4^
2. Blinding: ⁪patient^1^ ⁪investigator^2^ ⁪central committee^3^ ⁪open^4^ ⁪not stated^5^
3. Number of arms: ⁪ 2 ⁪ 3 ⁪ other________
4. Intervention: ⁪ CT vs P/O^1^ ⁪ CTa vs CTb^2^ ⁪ CTa vs CTab^3^ ⁪ H vs P/O^4^ ⁪ Ha vs Hb^5^ ⁪ CT vs H^6^ ⁪ TA vs P/O^7^ ⁪ CT vs CT/TA^8^ ⁪ RTa vs RTb^9^ ⁪ RT vs RT/CT^10^ ⁪ RT vs RT/HT^11^ ⁪ RT vs RT/TA^12^ ⁪ Sa vs Sb^13^ ⁪ S vs. S/RT^14^ ⁪ RT vs. RT/S^15^ ⁪ S vs S/CT^16^ ⁪ S vs S/HT^17^ ⁪ S vs S/TA^18^ ⁪ RT vs P/O^19^ ⁪ other^20^ __________
5. Sample size: Randomized______ Ineligible_____ Reasons for ineligible? ⁪ yes^1^ ⁪ no^2^
6. Eligible study population: ⁪ general^1^ ⁪ enriched^2^
7. Early termination: yes^1^ no^2^ Reasons ACC^1^ INT^2^ other^3^ __________

ITT Analysis: ⁪ all randomized^1^ ⁪ eligible only^2^ ⁪ not ITT^3^ ⁪ not stated^4^

⁪ explicit^5^ ⁪ implied^6^

1. Median F/U: ______ ⁪ not stated
2. Time of accrual: Start ________ Stop________ ⁪ not stated
3. Multicentre ⁪ yes^1^ ⁪ no^2^ ⁪ not stated^3^
4. Participants: ⁪ national^1^ ⁪ international^2^ ⁪ co-operative group^3^
5. Sponsorship ⁪ for profit^1^ ⁪ non-profit^2^ ⁪ mixed^3^ ⁪ not known^4^

Source(s) ⁪ govt^1^ ⁪ industry^2^ ⁪ foundation^3^ ⁪ co-op group^4^ ⁪other_____

Was drug provided by company? ⁪ yes^1^ ⁪ no^2^ ⁪ not stated^3^ ⁪ N/A^4^

## Reporting of Adverse Events (AE)

1. Were AE reported in paper?  Yes^1^ No^2^

2. Were AE reported in table/figure?  Yes^1^ No^2^

3. Were AE reported per type of event (list of events)?  Yes^1^ No^2^

4. If yes, how reported  Number^1^  Percent^2^  Mean^3^  Median^4^  Generic statement^5^

5. In which intervention group were AE reported?  Globally^1^  Per arm^2^  In only one arm^3^

6. Were statistical comparisons reported for each event?  Yes^1^  No^2^

7. Was threshold defined for AE reporting (i.e. >3% in any group)?  Yes^1^  No^2^ Value:_____

8. Were expected and unexpected events separated?  Yes^1^  No^2^

9. Were AE reported per patient?  Yes^1^  No^2^

10. If yes how  Globally^1^ (>=1 AE/patient)  For each event^2^  Other^3^

11. If yes, how reported  Number^1^  Percent^2^  Mean^3^  Median^4^  Generic statement^5^

12. In which intervention group was it reported?  Globally^1^  Per arm^2^  In only one arm^3^

13. Were AE reported per organ/system (i.e. gastrointestinal AE)?  Yes^1^ No^2^

14. If yes, how reported  Number^1^  Percent^2^  Mean^3^  Median^4^  Generic statement^5^

15. In which intervention group was it reported?  Globally^1^  Per arm^2^  In only one arm^3^

**C. Reporting of severity (SEV)**

1. Was information on severity of AE reported?  Yes^1^ No^2^

2. If yes, how reported  Number^1^  Percent^2^  Mean^3^  Median^4^  Generic statement^5^

3. In which intervention group was severity reported?  Globally^1^  Per arm^2^  In only one arm^3^

4. Was a definition of severity given?  Yes^1^ No^2^

5. Was a scale for severity grading used?  Yes^1^  No^2^

6. If yes, name of scale:  NCI CTCAE version 1 2 3 4  Other^0^ __________  Not reported^3^

7. Were only severe AE reported (death, life-threatening AE)? Yes^1^  No^2^

8. Did deaths due to AE occur? Yes^1^ No^2^  Not reported^3^

**D. Reporting of withdrawals due to AE (WTHDRL)**

1. Did withdrawals occur? Yes^1^ No^2^  Not reported^3^

2. Were withdrawals due to AE reported? Yes^1^  No^2^   unclear^3^

3. If yes, how reported  Number^1^  Percent^2^  Mean^3^  Median^4^  Generic statement^5^

4. In which intervention group were they reported?  Globally^1^  Per arm^2^  only one arm^3^

5. Were withdrawals reported for each specific AE?  Yes^1^  No^2^

6. Were withdrawals reported by severity (i.e. death, hospitalization)?  Yes^1^  No^2^

**E.** **Reporting of need for treatment discontinuation (TD)**

1. Did treatment discontinuations occur? Yes^1^ No^2^  Not reported^3^

2. Were reasons for treatment discontinuation reported?  Yes^1^  No^2^

3. If yes, were any due to AE?  Yes^1^ No^2^ unclear^3^

4. If yes, how reported  Number^1^  Percent^2^  Mean^3^  Median^4^  Generic statement^5^

5. If yes, in which intervention group  Globally^1^   Per arm^2^   In only one arm^3^

**F. Reporting of need for dose reduction (DR)**

1. Did dose reductions occur? Yes^1^ No^2^  Not reported^3^

2. Were reasons for dose reduction reported?  Yes^1^  No^2^

3. If yes, were any due to AE?  Yes^1^ No^2^ unclear^3^

4. If yes, how reported  Number^1^  Percent^2^  Mean^3^  Median^4^  Generic statement^5^

5. If yes, in which intervention group  Globally^1^   Per arm^2^   In only one arm^3^

**G. Statistical Analysis of AE (STAT)**

1. Were statistical tests for AE reported in paper?  Yes^1^  No^2^

- (a) Were statistical tests reported in a table/figure?  Yes^1^  No^2^

- (b) If so, were p values reported in table/figure?  Yes^1^  No^2^

2. Were p values for statistical analysis of AE reported in text?  Yes^1^  No^2^

3. Was statistical data on AE given in number of events?  Yes^1^  No^2^

4. Was statistical data on AE given in number of patients?  Yes^1^  No^2^

5. Were only AE with a specific p value reported?  Yes^1^  No^2^ P value |__|.|__|__|__|­

6. Was global comparison of intervention groups reported (global test)?

- (a) For total number of events  Yes^1^  No^2^
- (b) For total number of patients  Yes^1^  No^2^
- (c) For severity  Yes^1^  No^2^
- (d) For organ/system  Yes^1^   No^2^

7. Did analysis for assessing AE respect the ITT principle?  Yes^1^  No^2^

8. Was a safety analysis conducted (treated population only)?  Yes^1^  No^2^

9. Was sub-group analysis reported?  Yes^1^  No^2^
