# Supplementary material for: Quality of adverse event reporting in phase III randomized controlled trials of breast and colorectal cancer: A systematic review
Source: Cancer Med. 2020 May 26;9(14):5035–50. doi: 10.1002/cam4.3095 (PMC7367648; doi:10.1002/cam4.3095)
Supplement: Supplementary file 3 — Data S3 [file CAM4-9-5035-s003.docx]

**CONSORT AE Reporting – Supplementary Data S.3 Excluded Studies**

| **Study ID** | **Study Name** | **Reason for Exclusion** | **Citation Tracking** |
| --- | --- | --- | --- |
| Aparicio 2016 (1) | PRODIGE 34-FFCD 1402-ADAGE | Study protocol | No subsequently published report |
| Arjona-Sánchez 2018 | HIPECT 4 | Study protocol | No subsequently published report |
| Avallone 2016 | OBELICS | Study protocol | No subsequently published report |
| Barrett-Lee 2014 | ZICE | Supportive therapy for metastatic disease | n/a |
| Bear 2015 | NSABP B-40 | Duplicate | Original report published 2012 (see included studies) |
| BIG 1-98 2009 | BIG 1-98 | Duplicate | Original report published 2005 (see included studies) |
| Boccardo 2005 | ITA | Publication date (2005, pre-Nov) | This report is original report |
| Cartenì 2006 | n/a | Supportive therapy for metastatic disease | n/a |
| Cremolini 2017 | TRIBE-2 | Study protocol | No subsequently published report |
| Cristofanilli 2016 | PALOMA-3 | Duplicate | Original report published 2015 (see included studies) |
| de Azambuja 2014 | NeoALTTO | Duplicate | Original report published 2012 (see included studies) |
| Gligorov 2017 | SafeHer | Trial comparing device delivery methods for trastuzumab | n/a |
| Gnant 2015 | ABCSG-18 | Supportive therapy for metastatic disease | n/a |
| Goldhirsch 2013 | HERA | Duplicate | Original report published 2005 (see included studies) |
| Joensuu 2017 | FinXX | Duplicate | Original report published 2009 (see included studies) |
| Kurebayashi 2017 | NCT01546649 | Dosage trial | n/a |
| Leung 2017 | n/a | HIPEC treatment for metastatic disease | n/a |
| Loibl 2017 | PALOMA-3 | Duplicate | Original report published 2015 (see included studies) |
| Martin 2017 | BELLE-4 | Phase II/III study terminated at phase II | n/a |
| Miller 2005 | n/a | Publication date (2005, pre-Nov) | This report is original report |
| Mouridsen 2009 | BIG 1-98 | Duplicate | Original report published 2005 (see included studies) |
| Mukai 2010 | SELECT BC | Study protocol | Original report published 2016 (see included studies) |
| O'Shaughnessy 2016 | BCA 2001 | Phase II study | No subsequently published report |
| Ohue 2017 | PRECIOUS | Study protocol | No subsequently published report |
| Piccart-Gebhart 2005 | HERA | Publication date (2005, pre-Nov) | This report is original report |
| Pivot 2015 | CEREBEL | Primary endpoint concerning specific-site metastasis | n/a |
| Sclafani 2015 | MK-0646-004 | Trial terminated at phase II | n/a |
| Sclafani 2017 | MK-0646-004 | Trial terminated at phase II | n/a |
| Snoeren 2017 | HEPATICA | Primary endpoint concerning specific-site metastasis | n/a |
| Steger 2007 | ABCSG-14 | Dosage trial | n/a |
| Taieb 2017 | PETACC-8 | Duplicate | Original report published 2014 (see included studies) |
| Trédan 2015 | CA163-139 | Phase II study | No subsequently published report |
| Vogel 2001 | n/a | Dosage trial | n/a |
| von Minckwitz 2008 | GeparTrio | Dosage trial | n/a |
| Yamamoto 2015 | JBCRN-05 | Phase II study | No subsequently published report |
